# The Alleviative Effects of *Weizmannia coagulans* CGMCC 9951 on the Reproductive Toxicity of *Caenorhabditis elegans* Induced by Polystyrene Microplastics

**DOI:** 10.3390/microorganisms13030497

**Published:** 2025-02-24

**Authors:** Chengmei Li, Lina Zhao, Jiajia Fan, Wentong Qi, Xuan Li, Yuwan Li, Pingping Tian, Ying Wu, Shaobin Gu

**Affiliations:** 1 College of Food and Bioengineering, Henan University of Science and Technology, Luoyang 471000, China; 2 Henan Engineering Research Center of Food Microbiology, Luoyang 471000, China; 3 National Demonstration Center for Experimental Food Processing and Safety Education, Luoyang 471000, China

**Keywords:** polystyrene microplastics, reproductive toxicity, *Weizmannia coagulans* CGMCC 9951, *Caenorhabditis elegans*

## Abstract

The increased emission and accumulation of microplastics pose a severe threat to humans and the environment. As effective biological agents for alleviating the effects of microplastics, the mechanism of action of probiotics remains unclear. In this study, based on the successful establishment of a reproductive virulence model of *Caenorhabditis elegans* (*C. elegans*), we explored the effect and mechanism of *Weizmannia coagulans* CGMCC 9951 (*W. coagulans* CGMCC 9951) on the reproductive toxicity of *C. elegans*. Our results showed that the gonad area and the number of offspring increased but the number of germ cells undergoing apoptosis decreased by 14% and 24% in *C. elegans*, after CGMCC 9951 treatments. Antioxidant test results showed that CGMCC 9951 increased the activity of Superoxide Dismutase (SOD), Catalase (CAT), and the content of Glutathione (GSH) in *C. elegans*. In addition, it was found by qPCR and mutagenesis experiments verified that CGMCC 9951 alleviated reproductive toxicity through the DNA checkpoint signaling pathway. Our findings suggested that CGMCC 9951 could alleviate the reproductive toxicity of polystyrene microplastics in *C. elegans* by enhancing antioxidant capacity and inhibiting DNA damage checkpoint signaling pathway. The above results suggest that probiotics can be used as a potential approach to alleviate the reproductive toxicity induced by polystyrene microplastics in humans.

## 1. Introduction

Microplastics (MPs) are usually the result of the disintegration of larger plastic waste, including vehicle tires, synthetic clothing, and drug vectors [1,2]. Microplastics have been found in oceans and wildlife worldwide because of the rapid growth in plastic production worldwide over the past 60 years [3,4,5]. The 80 nm MP water exposure could cause severe damage to intestinal villi, imbalance of intestinal flora homeostasis, significant liver steatosis, and lipid metabolism disorder in tilapia. Microplastics are posing a serious threat to human health [6,7,8]. It has been reported that probiotics or probiotics combined with melatonin or polysaccharide can alleviate the reproductive toxicity and blood toxicity of polystyrene-exposed mice, and the sperm quality of zebrafish [9]. However, little is known about the toxic effects of microplastics and how to mitigate their adverse effects.

It has been found that probiotics (*Lactobacillus*, *Bifidobacterium longum*) can alleviate the toxicity of polystyrene microplastics (PS-MPs), reduce the inflammatory response of PS-MPs exposure, and then alleviate the sperm quality of male mice exposed to PS-MPs [10]. *Weizmannia coagulans* CGMCC 9951 (*W. coagulans* CGMCC 9951), isolated from the feces of healthy piglets, exhibits good probiotic properties and has potential probiotic attributes. It can survive and colonize the gastrointestinal tract and exert beneficial effects. *W. coagulans* CGMCC 9951 had good antibacterial activity against various pathogenic bacteria [11]. *W. coagulans* can inhibit the growth of harmful bacteria and increase the number of beneficial bacteria, which improves digestion ability and immunity, playing a role in regulating human health [12,13]. *W. coagulans* Unique IS2 significantly decreased the symptoms of constipation [14]. *W. coagulans* BC99 influences the production of valeric acid by regulating the gut microbiota, thereby alleviating inflammation and oxidative stress responses in mice infected with Helicobacter pylori [15]. The effects of probiotics are strain-specific. As *W. coagulans* appears to have the capability to improve various aspects of toxicity, it was, therefore, valuable to investigate the potential impact of *W. coagulans* CGMCC 9951 in mitigating polystyrene microplastic (PS-MPs, PS) toxicity.

*Caenorhabditis elegans* (*C. elegans*) is one of the animal models for toxicity evaluation of different toxicants found in the environment, like nanomaterials and heavy metals [16,17,18,19]. Some researchers have discovered that exposure to microplastics can result in oxidative stress in animals, leading to detrimental effects on their reproductive, respiratory, endocrine, circulatory, and central nervous systems [20,21]. For example, water pollution from phosphamide factories or urinary phthalate metabolites increased oxidative stress and DNA damage, and caused developmental and reproductive toxicity [22,23]. A recent study showed that paeoniflorin alleviated the reproductive toxicity caused by polystyrene exposure through the reduction in the expression of genes involved in DNA damage checkpoints [24]. We were intrigued to explore whether *W. coagulans* CGMCC 9951 could likewise mitigate the reproductive toxicity induced by microplastics. Our focus was on determining if it could achieve this by modulating the DNA damage checkpoint pathway within *C. elegans*.

In the present study, we aimed to investigate the beneficial influence of *W. coagulans* CGMCC 9951 against PS-induced reproductive toxicity in *C. elegans* and the underlying mechanisms. Our results showed that condensation of *W. coagulans* CGMCC 9951 mitigated the reproductive toxicity of PS exposed to *C. elegans*. This study provided new insights into mitigating the reproductive toxicity of microplastics in mammals. Moreover, our data suggested that oxidative stress and DNA damage checkpoint signaling pathways played important roles in *W. coagulans* CGMCC 9951’s mitigation of reproductive toxicity in microplastic-exposed nematodes. These findings provide a novel approach to mitigate the biological reproductive toxicity of polystyrene exposure.

## 2. Materials and Methods

### 2.1. Exposure and Strains Maintenance

*W. coagulans* CGMCC 9951 was deposited in the General Microbiology Center of China Microbiological Culture Preservation Management Committee (Beijing, China, Preservation number: CGMCC NO. 9951). It is isolated from the feces of healthy piglets and possesses good probiotic properties, which include potential probiotic attributes [11]. The wild-type (N2), transgenic *C. elegans* WS1433/[HUS-1::GFP], and MT4770/[ced-9(n1950)] were obtained from the Caenorhabditis Genetics Center (CGC, University of Minnesota, Saint Paul, MN, USA) in this study. We utilized the WS1433 strain to observe germline DNA damage. Low-copy integrated array of 1144 bp *hus-1* promoter and genomic coding sequence fused to GFP. Fluorescence intensity represents the expression level of this gene [24]. MT4770 represents the ced-9 (n1950) gain of function mutants [25]. All *C. elegans* strains were grown on nematode growth media (NGM) agar media containing *Escherichia coli* OP50 (*E. coli* OP50) and kept at 20 °C in darkness [26,27]. Pregnant *C. elegans* were washed and lysed by adding Clorox solution (0.45 M NaOH and 2% HOCl) to a centrifuge tube containing the sediment. Embryos obtained from adult nematodes treated with bleach were incubated in M9 buffer overnight (0.3 g KH_2_PO_4_, 0.6 g Na_2_HPO_4_, 0.5 g NaCl, 0.1ml 1M MgSO_4_, 100 mL H_2_O) [24]. We conducted experiments on L1-stage nematodes by exposing them to microplastics.

Two kinds of PS-MP particles were selected for study (Tianjin Saierqun Technology Co., Ltd., Tianjin, China). One was polystyrene microplastic particles (0.6~1 μm) for the toxicity test. The other was Red Fluorescent Polystyrene Microspheres (1 μm), which were used to observe the accumulation of microplastic particles in *C. elegans*. The polystyrene stock solution was centrifuged, the supernatant was removed, and the polystyrene pellets were soaked in 75% ethanol. After centrifugation, the polystyrene pellets were resuspended in sterile water and then stored at 4 °C. In this study, sterile water was utilized to formulate the diluent for PS exposure concentration (0.01, 0.1, 1 mg/mL), which was thoroughly blended with *E. coli* OP50 and applied to NGM medium in a 60 mm Petri dish [28]. The plates were then incubated at 20 °C overnight for future use.

### 2.2. Establish a Reproductive Toxicity Model of Microplastics

In this study, *W. coagulans* CGMCC 9951 is similar to *E. coli* OP50 and serves as food for *C. elegans.* We exposed the synchronized L1 stage nematodes to red fluorescent microspheres (0, 0.01, and 1 mg/mL) or polystyrene microspheres (0, 0.01, 0.1, and 1 mg/mL) for 72 h [29].

#### 2.2.1. Effect of Fluorescent Microspheres Accumulation of Different Doses of Microplastics in *C. elegans*

As the control group, NGM plates with only *E. coli* OP50 were utilized. For the exposure groups, NGM plates incorporating different concentrations of microplastics along with *E. coli* OP50 were employed. The synchronized L1 stage nematodes were exposed to red fluorescent microspheres (excluding the control group with no red fluorescent microspheres, and concentrations of 0.01 and 1 mg/mL) for 72 h. Nematodes were washed three times with M9 buffer and measured and photographed by fluorescence microscope (Leica DM2500, Leica, Germany) within 2 h after the end of exposure [30]. At least three separate trials were conducted, with thirty nematodes assessed in each group.

#### 2.2.2. Effect of Body Length on Nematodes at Different Doses of Microplastics

After 72 h, *C. elegans* were washed three times using M9 buffer, and we measured the body length with the aid of an optical microscope within 2 h of the exposure period ending [31,32].

#### 2.2.3. Effect of Survival Rate on Nematodes at Different Doses of Microplastics

After 72 h, *C. elegans* were moved daily to fresh NGM agar media containing *E. coli* OP50 (as the control group) or polystyrene microspheres and *E. coli* OP50 (as the PS group) for the first 7 days [31]. The sign of death in *C. elegans* was its lack of response to contact with a pick needle. The survival rate was assessed by calculating the percentage of surviving nematodes.

### 2.3. Effects of W. coagulans CGMCC 9951 Against Reproductive Toxicity of Microplastics

In *C. elegans*, lots of sublethal endpoints have already been applied to evaluate the reproductive toxicity of specific toxicants, including germline apoptosis and brood size [33,34]. Nematodes were moved to fresh NGM plates daily, and the nematode count was recorded until egg laying ceased. Three independent experiments were conducted, with thirty nematodes scored for each group. *C. elegans* were stained with acridine orange solution (AO, 25 μg/mL) for 2 h [35]. Following staining, the upper layer of AO solution was removed, and the nematodes were placed on NGM (supplemented with *E. coli* OP50) and allowed to recover for 60 min at 20 °C, facilitating the expulsion of excess AO solution from the intestine. The cells undergoing apoptosis exhibited a yellow-green hue following AO staining, indicating heightened DNA fragmentation, while undamaged cells displayed a consistent green coloration [36]. At least three separate trials were conducted, with thirty nematodes assessed in each group. According to earlier studies, the area of the gonad arm represents variations in gonad development [35]. The gonad area of *C. elegans* was calculated with the aid of an optical microscope.

### 2.4. Effect of W. coagulans CGMCC 9951 on Oxidative Stress in Nematodes Exposed to Microplastics

Synchronized L1-stage nematodes were transferred to PS (1 mg/mL) NGM plates and normal NGM plates (only *E. coli* OP50, control group). After 48 h, nematodes on microplastic plates were transferred to normal NGM plates and NGM plates containing different concentrations of *W. coagulans* CGMCC 9951 (10^7^, 10^8^, 10^9^ cfu/mL), which were used as model and probiotic treatment groups, respectively. The groups of *W. coagulans* CGMCC 9951 with bacterial liquid concentrations of 10^7^, 10^8,^ and 10^9^ cfu/mL are denoted as the PS + L group, the PS + M group, and the PS + H group. However, control nematodes were also placed on regular NGM plates. Once the nematodes reached the L4 stage, they were harvested using M9 buffer. The *C. elegans* at the L4 stage were relatively large in size. Their body length has increased significantly compared to the previous larval stages. They were slender and transparent, enabling clear observation of their internal structures [37].

In nematodes, levels of intestinal ROS can be used to reflect the induction of oxidative stress [38]. Given that reactive oxygen species (ROS), the primary compounds produced during oxidative stress in the body, have been acknowledged as crucial controllers in the process of apoptosis [39], 5′,6′- chloromethyl-2,7-dichlorodihydrofluorescein diacetate (H_2_DCFDA, 10 μM) was used to measure the concentration of ROS in *C. elegans* (Shanghai Aladdin Biochemical Technology Co., Ltd., Shanghai, China). Nematodes were collected using M9 buffer solution and then underwent three rounds of purification with M9 buffer. Subsequently, 200 μL of 10 mM ROS probe solution was added in a light-free environment, thoroughly mixed, and incubated at 20 °C for 2 h. Afterward, nematodes were washed three times with M9 buffer to remove excess dye. Finally, the nematodes were placed on a slide and observed using a fluorescence microscope within a 2 h period [38,40]. The Image J software (version number: v1.54 m) from the National Institutes of Health in the USA was employed for the analysis of ROS fluorescence intensity.

In addition, antioxidant indexes, such as superoxide enzyme (SOD) activity, catalase (CAT) activity and glutathione (GSH) content, which were detected by using total SOD activity detection kit, reduced CAT kit and GSH assay kit (Nanjing Jiancheng Bioengineering Research Institute Co., Ltd., Nanjing China), respectively.

### 2.5. Quantitative Real-Time PCR

Ribonucleic Acid (RNA) was extracted from *C. elegans* using a microsample total RNA extraction kit (Novozymes Investment Co., Ltd., Beijing, China) and reverse transcribed with the kit (Beijing Kang Runcheng Industry Biotechnology Co., Ltd., Beijing, China). The RNAs were evaluated for their quality using 1% agarose gel, and the expression of key genes was analyzed using 2× RealStar Fast SYBR qPCR mix through a CFX96 Touch Real-Time PCR instrument. Data analysis was conducted using the 2^−∆∆Ct^ as described [41]. The reaction conditions were as follows: 95 °C for 30 s, 40 cycles of 95 °C for 5 s, and 60 °C for 30 s. At least three independent experiments were performed. Table S1 displayed the primer details utilized in the Real-Time PCR.

### 2.6. Effects of W. coagulans CGMCC 9951 on Apoptosis and DNA Damage in Germ Cells

DNA damage can be detected using transgenic *C. elegans* WS1433, which carries the HUS-1::GFP fusion protein. The fluorescence intensity of HUS-1::GFP (WS1433) was observed using a fluorescence microscope. Additionally, the amount of apoptotic germ cells in MT4770 mutants was examined with a fluorescence microscope.

### 2.7. Statistical Analysis

Mean ± SD was used to express all data. Graphs were performed with Origin2023 software (version number: 10.0). We determined statistical significance by *t*-test and one-way ANOVA using GraphPad Prism software (9.5.1). At least three separate trials were conducted, with thirty nematodes assessed in each group.

## 3. Results

### 3.1. Establishment of a Reproductive Toxicity Model of Microplastics in C. elegans

**Figure 1:**
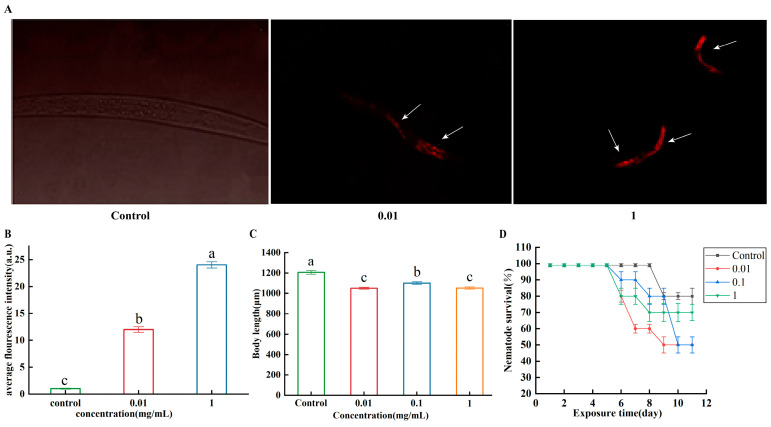
A nematode microplastic toxicity model was established. (**A**) Fluorescence images of nematodes. Arrows indicate PS-MPs. (**B**) Comparison of the effect of accumulation of PS-MPs in nematodes. (**C**) Effect of PS-MPs treatment on the body length. (**D**) Effect of PS-MPs treatment on the survival rate of *C. elegans* exposed to different doses of PS-MPs. ^abc^: Means not sharing a common letter are significantly different among the groups at *p* < 0.05.

The accumulation of different doses of microplastics in *C. elegans* was studied. The fluorescence intensity of the 1 and 0.01 mg/mL exposure group was significantly higher than that of the control group (Figure 1A,B). The results suggested that higher concentrations of microplastics lead to greater accumulation in nematodes and adverse effects on nematodes. PS did cause significant reduction of 12.8%, 8.7%, and 12.7% in body length, after exposure to PS-MPs (0.01, 0.1, 1 mg/mL) (Figure 1C). PS-MPs (0.01, 0.1 mg/mL) resulted in a significant reduction in survival rate compared with control on day 11 (Figure 1D). Seventy percent of the nematodes in PS-MPs (1 mg/mL) were still alive on day 11.

### 3.2. W. coagulans CGMCC 9951 Treatment Affected the Reproductive Ability

#### 3.2.1. Effect of *W. coagulans* on the Number of Offspring of Nematodes Exposed to Microplastics

**Figure 2:**
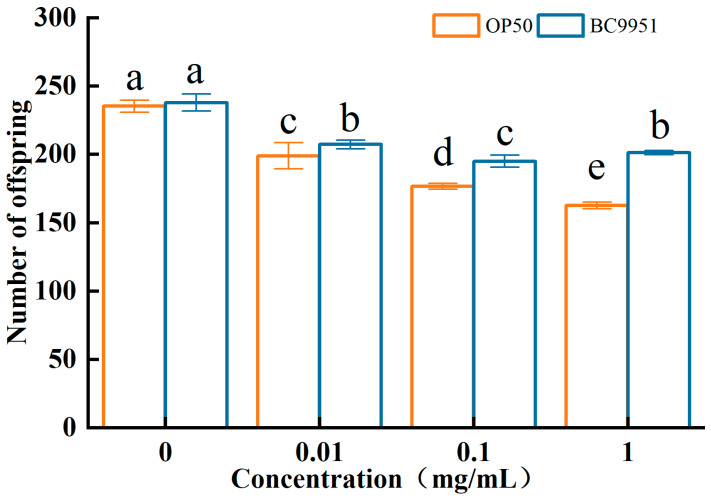
Effect of *W. coagulans* treatment on brood size of nematodes exposed to different doses of PS-MPs. ^abcde^: Means not sharing a common letter are significantly different among the groups at *p* < 0.05.

Endpoints of brood size were commonly used to assess the reproductive capacity of nematodes. As illustrated in Figure 2, acute exposure to PS significantly affected the brood size in nematodes. The number of offspring decreased with the increase in microplastic concentration. There was no significant difference between *E. coli* OP50 and *W. coagulans* CGMCC 9951 exposed to microplastics. However, when the microplastic exposure concentration was 0.01, 0.1, and 1 mg/mL, *W. coagulans* CGMCC 9951 groups increased by 4.2%, 10.4%, and 23.8% over *E. coli* OP50 groups. When the concentration of microplastic exposure reached 1 mg/mL, the effectiveness of toxicity mitigation was enhanced.

#### 3.2.2. Effect of *W. coagulans* on Germ Cell Apoptosis of Nematodes Exposed to Microplastics

**Figure 3:**
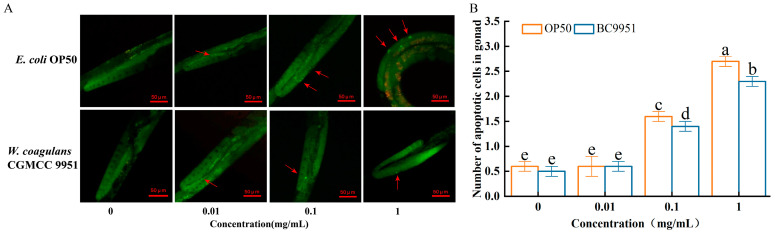
Effect of *W. coagulans* treatment on germline apoptosis. (**A**) The arrowhead symbolizes the apoptotic signals of the germline. (**B**) Comparison of the effect of PS-MPs on the cell corpse per gonad in *C. elegans.* ^abcde^: Means not sharing a common letter are significantly different among the groups at *p* < 0.05.

Behind the PS-MPs (0.1, 1 mg/mL) exposure, the number of apoptotic gonads in *C. elegans* (*W. coagulans* CGMCC 9951 groups) decreased by 12.5% and 14.8% compared with the control group (fed with *E. coli* OP50) (Figure 3). However, there was no significant difference between *W. coagulans* groups and *E. coli* OP50 when PS was not exposed and exposed to 0.01 mg/mL in *C. elegans*.

#### 3.2.3. Effect of *W. coagulans* on Gonad Area of Nematodes Exposed to Microplastics

**Figure 4:**
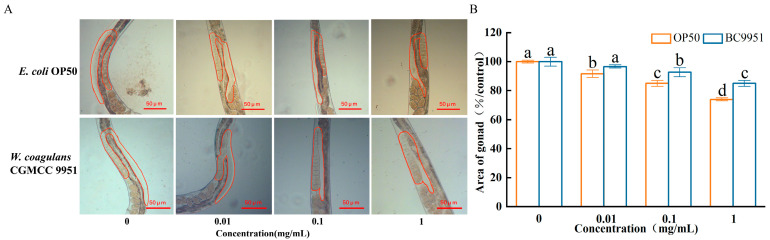
Effect of *W. coagulans* treatment on the gonad area. (**A**) The area of the unilateral gonad of the nematode. (**B**) Comparison of the effect of PS-MPs on the gonad area. ^abc^: Means not sharing a common letter are significantly different among the groups at *p* < 0.05.

When nematodes were fed with *E. coli* OP50, the gonad area of PS-MPs (0.01, 0.1, 1 mg/mL) groups was reduced significantly, compared with the control group (Figure 4). When nematodes were fed with *W. coagulans* CGMCC 9951, the gonad area of *C. elegans* increased by 5.5%, 9%, and 14.9% in MPs groups (0.01, 0.1, 1 mg/mL), compared with *E. coli* OP50 groups. *W. coagulans* had a better mitigation effect on the reproductive toxicity of *C. elegans* exposed to high concentrations of microplastics.

### 3.3. W. coagulans CGMCC 9951 Improves Antioxidant Capacity to Alleviate the Toxicity of Microplastics

**Figure 5:**
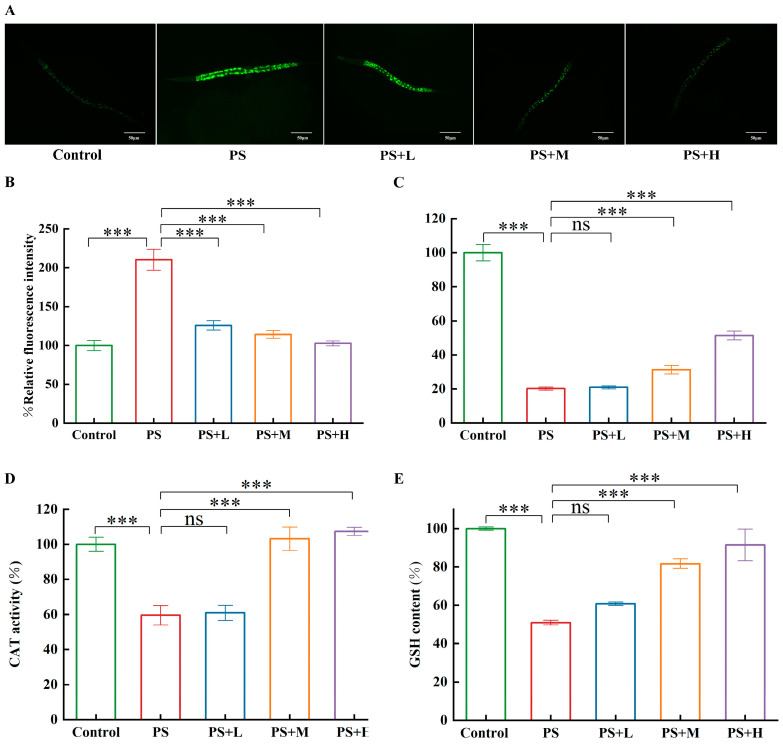
*W. coagulans* CGMCC 9951 could improve the antioxidant capacity of nematodes exposed to PS-MPs. (**A**) Effect of *W. coagulans* CGMCC 9951 on ROS content in PS-MPs-exposed *C. elegans*. (**B**) Relative expression levels of ROS. (**C**) Effect of *W. coagulans* CGMCC 9951 on SOD activity in *C. elegans*. (**D**) Effect of *W. coagulans* CGMCC 9951 on CAT activity in *C. elegans*. (**E**) Effect of *W. coagulans* CGMCC 9951 on GSH content in *C. elegans*. *** *p* < 0.001; ns, no significant difference.

To examine the impact of the oxidative stress response on alleviating PS-MP toxicity in *C. elegans*, we assessed the levels of reactive oxygen species (ROS), as well as the functionality of antioxidant enzymes and the concentration of antioxidants. Compared with the control group, the fluorescence intensity of *W. coagulans* CGMCC 9951 treatment groups decreased (Figure 5A). Quantitative data showed that PS-MP exposure induced a 1-fold increase in ROS levels compared with the control group. However, compared with PS group, *W. coagulans* CGMCC 9951 (10^7^, 10^8^, 10^9^ cfu/mL) significantly reduced ROS levels by 40%, 45%, and 51%, respectively (Figure 5B). The GSH content, SOD activity, and catalase activity in PS-MPs groups nematodes were reduced, respectively, in *C. elegans* (Figure 5C–E), in comparison with the control group. *W. coagulans* CGMCC 9951 (10^8^, 10^9^ cfu/mL) treatment groups had a significant increase in SOD activity, CAT activity, and GSH content.

### 3.4. Effects of W. coagulans CGMCC 9951 Treatment Against PS-MPs Toxicity in Inducing Germline Apoptosis and DNA Damage

#### 3.4.1. Effects of *W. coagulans* Treatment on Expressions of Genes Governing Germline Apoptosis and DNA Damage in MP-Exposed Nematodes

**Figure 6:**
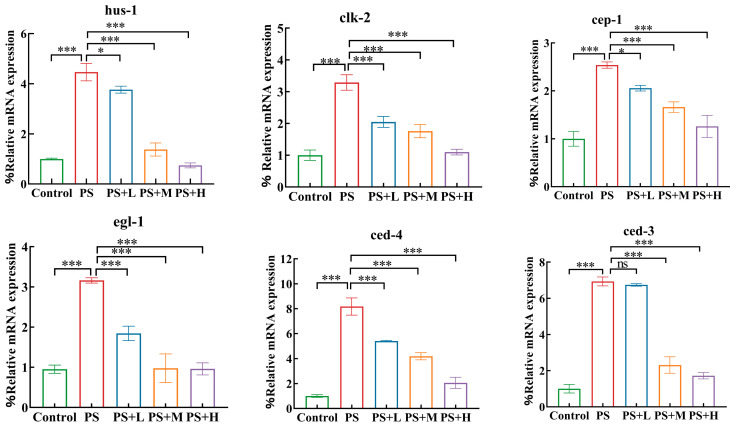
Effect of *W. coagulans* CGMCC 9951 treatment on gene expression controlling apoptosis in germlines. * *p* < 0.05, *** *p* < 0.001; ns, no significant difference.

CLK-2and HUS-1 form the essential molecular apparatus for germline DNA damage checkpoint in *C. elegans* [24]. The levels of *clk-2* and *hus-1* were markedly elevated after exposure to 1 mg/mL PS-MPs. Exposure to 1 mg/mL PS resulted in a significant increase in the expressions of *cep-1*, *clk-2*, and *hus-1* (Figure 6). Subsequent treatment with *W. coagulans* after exposure to 1 mg/mL PS further significantly decreased the expression of these three genes compared to that in nematodes exposed to PS.

CED-3 and CED-4 play a central role in the process of apoptosis in *C. elegans*, and their interaction and regulatory mechanisms are essential for cell death [24]. Exposure to 1 mg/mL PS led to a significant increase in the expressions of *ced-3*, *ced-4*, and *egl-1* compared to the control group (Figure 6). Subsequent treatment with 10^7^, 10^8^, and 10^9^ cfu/mL *W. coagulans* in nematodes exposed to 1 mg/mL PS significantly decreased the expressions of *egl-1*, *ced-4*, and *ced-3* compared to those in PS-MPs exposed nematodes.

#### 3.4.2. Validation of Nematode Mutants

**Figure 7:**
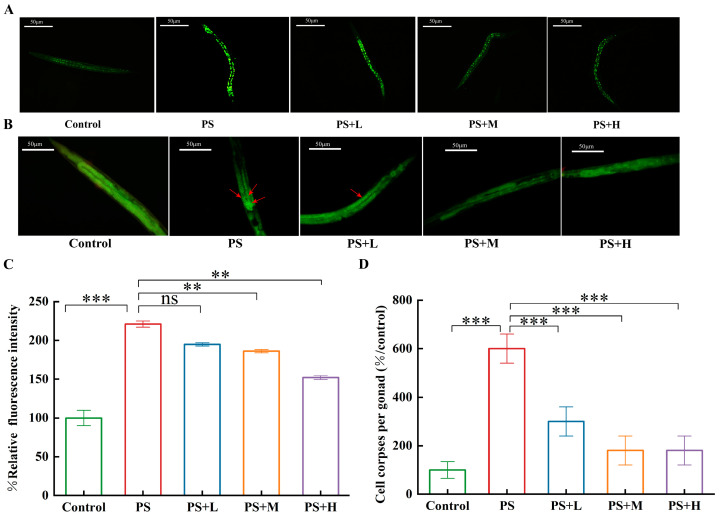
Validation experiments were performed with mutant nematodes. (**A**) The comparison of HUS-1::GFP in nematodes exposed to PS-MPs. (**B**) AO staining images. The arrowhead serves to indicate the germline apoptotic signals. (**C**) The quantification of HUS-1::GFP. (**D**) The relative number of germ cell corpses.** *p* < 0.01, *** *p* < 0.001; ns, no significant difference.

We used WS1433 strains to examine the expression level of the *hus-1* gene, and WS1433 expressed HUS-1::GFP. The result indicated that exposure to PS-MPs resulted in elevated GFP fluorescence signal in comparison to control. In contrast, the fluorescence intensity in *C. elegans* treated with *W. coagulans* CGMCC 9951 decreased (Figure 7A,C).

CED-9 exerts its anti-apoptotic effect by preventing CED-4 from activating CED-3 by anchoring CED-4 to the cell membrane [24]. The number of apoptotic cells in MT4770 was significantly increased after PS-MP exposure compared to the control (Figure 7B,D). The number of dead nematode germ cells in the *W. coagulans* group was significantly reduced than that in the PS exposure group, which further verified the role of the *ced-9* gene in the alleviation of germ cell apoptosis in nematodes exposed to PS-MPs by *W. coagulans*.

## 4. Discussion

Microplastics and heavy metals are increasingly harmful to ecology, animals, and plants, and research methods are needed to combat this harm [42,43]. However, there are a few methods to alleviate microplastics with probiotics, and the mechanism of alleviating the toxicity is not clear. Instances of beneficial bacteria have been employed to treat diseases. Probiotics and polyphenols can prevent or slow the onset of cognitive impairment and neuroinflammation by inhibiting oxidative stress, and inflammatory pathways in mice [44,45]. *W. coagulans* BC99 enhances intestinal barrier function by modulating butyrate formation to alleviate acute alcohol intoxication in rats [46]. In *C. elegans*, *W. coagulans* BC99 effectively curbed hyperuricemia levels and diminished oxidative stress markers [47]. We investigated the mechanism by which *W. coagulans* CGMCC 9951 alleviated the reproductive toxicity of microplastic-exposed nematodes.

*W. coagulans* CGMCC 9951 is a novel strain isolated from healthy piglet feces, which has promising probiotic characteristics [11]. Previous research has indicated that the addition of probiotics has been effective in suppressing the activation of IL-17A signaling induced by gut microbes; thus, it alleviates the inflammatory response and ameliorates the decline in sperm quality caused by PS-MPs in mice [10]. We found that *W. coagulans* CGMCC 9951 could significantly decrease reproductive toxicity using the evaluation model. It decreased the number of apoptotic gonadal cells and the damage of gonads. The number of offspring of nematodes exposed to microplastics was increased after the *W. coagulans* CGMCC 9951 treatment. However, the mechanism by which *W. coagulans* alleviates the toxicity of PS remains unclear.

ROS level causes damage to DNA, protein, and lipids, which could lead to cell apoptosis or necrosis and a serious impact on human health [48,49,50]. Products of lipid peroxidation also bind with extracellular signal-regulated kinase, C-Jun N-Terminal Kinase, and p38 to activate mitogen-activated protein kinase (MAPK). JNK and p38 MAPK in the MAPK family play a key role in apoptosis. They can affect the apoptotic process by regulating the expression and function of apoptosis-related proteins [24]. Mitochondrial dysfunctions resulted in increased ROS production, aging, and apoptosis, or programmed cell death, and were closely associated with many pathologies, such as cancer, metabolic disorders, and type 2 diabetes. Moreover, ROS production increases at high mitochondrial membrane potential, and the excessive accumulation of ROS leads to oxidative stress, aging, and age-related diseases [51,52]. Excessive ROS formation is the cause of oxidative damage, which is affected by SOD and CAT [53]. In terms of mechanism, Longan polysaccharide can increase the activity of antioxidant enzymes, reduce lipid peroxidation, and enhance the body’s resistance to stress damage of nematodes [54]. Previous studies have found that microplastics can cause inflammation by causing oxidative stress in the body [55]. We investigated whether *W. coagulans* CGMCC 9951 alleviated microplastic toxicity through oxidative stress. In our study, we found that the content of ROS was significantly reduced in the *W. coagulans* group compared with the PS group. GSH is a small-molecule scavenger, and the level of GSH is a crucial indicator of the extent of antioxidant activity in nematodes [56]. *W. coagulans* CGMCC 9951 significantly increased the activity of CAT, SOD, and GSH content in PS-MPs-exposed nematodes (Figure 5), which improved the health level of nematodes. We found that *W. coagulans* CGMCC 9951 alleviated the toxicity of microplastics by improving antioxidant capacity. Next, we would like to know whether *W. coagulans* CGMCC 9951 could alleviate microplastic reproductive toxicity through other pathways.

**Figure 8:**
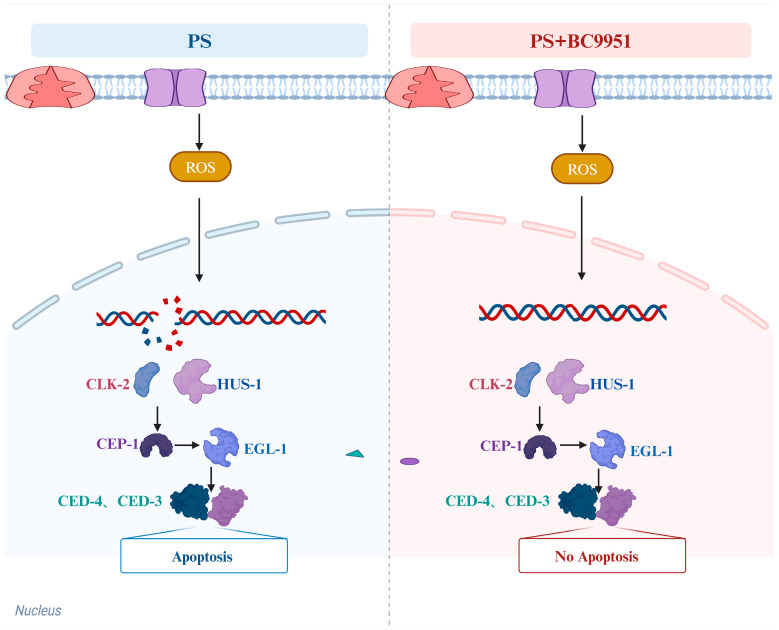
*W. coagulans* CGMCC 9951 alleviated the reproductive toxicity of microplastic exposure by inhibiting the DNA damage-induced apoptosis pathway in the nematode.

Previous studies have found that treatment with paeoniflorin may reduce the damaging impact of PS-NP on reproductive ability by decreasing the induction of germline apoptosis [24,57]. To further investigate whether *W. coagulans* CGMCC 9951 alleviated reproductive toxicity by alleviating apoptosis of nematode germ cells, the expression level of genes involved in the DNA damage checkpoint signaling pathway was measured. The DNA damage response is regarded as a crucial initiator of stress-induced apoptosis in the germ cells of *C. elegans* [58]. HUS-1 and CLK-2 are important checkpoint proteins in *C. elegans*, which transmit DNA damage signals to the core apoptotic pathway via CEP-1/p53 [24]. In *C. elegans*, CED-3 is an essential protein for cell death during development. CED-3 has sequence homology with the mammalian cysteine protease ICE, also known as caspase-1, suggesting that CED-3 is a key enzyme in executing cell death. CED-4, the mammalian equivalent of Apaf-1 in *C. elegans*, binds to the precursor form of CED-3, leading to CED-3 autoactivation and cell death. Furthermore, the interaction between CED-4 and CED-9 is essential for the regulation of CED-3 activity. CED-9 inhibits CED-3 activation by forming a complex with CED-4, thereby preventing cell death [59]. The results showed that *W. coagulans* CGMCC 9951 significantly decreased the expression of *hus-1*, *clk-2*, *cep-1*, *egl-1*, *ced-3*, and *ced-4* genes in nematode cells exposed to PS-MPs (Figure 6). This is consistent with the findings of Professor Wang [24]. The results indicated that as the concentration of *W. coagulans* CGMCC 9951 increased, its effect in mitigating the toxicity of microplastic exposure on *C. elegans* became more pronounced. Functional nutrients, among which *W. coagulans* CGMCC 9951 is included, exhibit hormetic dose-dependent effects. The WS1433 strain was used to detect the expression level of the *hus-1* gene, which was fused to a green fluorescent protein (GFP). This transgenic approach allowed the expression of the *hus-1* gene to be visualized by fluorescence microscopy to assess its activity and distribution in cells. CED-9 is a gene in *C. elegans* that encodes a functional protein homologous to mammalian BCL-2, which plays a protective role in cell survival. In mutant empirical studies, it was confirmed that *W. coagulans* CGMCC 9951 effectively reduces the apoptosis rate of germline cells by suppressing DNA damage (Figure 7). One potential scenario involves the activation of DNA checkpoint genes *hus-1* and *clk-2* by PS, which, in turn, triggers *cep-1* activation. This leads to an upregulation of *egl-1* expression and subsequently initiates downstream apoptotic signaling (Figure 8). Previous studies have found that probiotics alleviate male reproductive system toxicity in mice caused by polystyrene microplastics by reducing the inflammatory response [60,61]. Our findings significantly contribute to the research on probiotics’ role in alleviating the toxicity of microplastics in mice. *W. coagulans* CGMCC, as a functional food, has the potential to be applied in treating or preventing various diseases caused by microplastic exposure in humans in the future.

We are aware that there are some limitations to our study. The physiological mechanisms in *C. elegans* diverge substantially from those of mammals. It is possible that different species exposed to microplastics have different regulation ways of anti-oxidative stress and inhibiting DNA damage. We will repeat our *C. elegans* experiment in mammalian models to verify the effects of *W. coagulans* CGMCC 9951 on the reproductive toxicity caused by microplastic exposure in mammals. We aim to explore how *W. coagulans* 9951 mitigates microplastic toxicity in more complex organisms and compare these effects with our observations in *C. elegans*.

## 5. Conclusions

Through the established microplastic toxicity model, *W. coagulans* CGMCC 9951 was found to alleviate the reproductive toxicity of nematodes exposed to microplastics. Further studies showed that *W. coagulans* CGMCC 9951 alleviated the reproductive toxicity of microplastics by improving the antioxidant capacity and inhibiting the DNA damage checkpoint signaling pathway in *C. elegans*. The probiotic effects of *W. coagulans* CGMCC 9951 make it useful in the future as a means of mitigating the reproductive toxicity of environmental contaminants.

## Data Availability

The original contributions presented in this study are included in the article. Further inquiries can be directed to the corresponding author.

## Supplementary Materials

The following supporting information can be downloaded at: https://www.mdpi.com/article/10.3390/microorganisms13030497/s1, Table S1: Primer sequences for real-time PCR.

## References

[1] Browne M.A., Crump P., Niven S.J., Teuten E., Tonkin A., Galloway T., Thompson R. (2011). Accumulation of microplastic on shorelines worldwide: Sources and sinks. Environ. Sci. Technol..

[2] Wright S.L., Kelly F.J. (2017). Plastic and human health: A micro issue?. Environ. Sci. Technol..

[3] Eerkes-Medrano D., Thompson R.C., Aldridge D.C. (2015). Microplastics in freshwater systems: A review of the emerging threats, identification of knowledge gaps and prioritisation of research needs. Water Res..

[4] Hartmann N.B., Huffer T., Thompson R.C., Hassellov M., Verschoor A., Daugaard A.E., Rist S., Karlsson T., Brennholt N., Cole M. (2019). Are we speaking the same language? Recommendations for a definition and categorization framework for plastic debris. Environ. Sci. Technol..

[5] Rochman C.M., Browne M.A., Halpern B.S., Hentschel B.T., Hoh E., Karapanagioti H.K., Rios-Mendoza L.M., Takada H., Teh S., Thompson R.C. (2013). Policy: Classify plastic waste as hazardous. Nature.

[6] Abbasi S., Turner A. (2021). Human exposure to microplastics: A study in Iran. J. Hazard. Mater..

[7] Prata J.C., da Costa J.P., Lopes I., Duarte A.C., Rocha-Santos T. (2020). Environmental exposure to microplastics: An overview on possible human health effects. Sci. Total Environ..

[8] Vethaak A.D., Legler J. (2021). Microplastics and human health. Science.

[9] Zhang L., Jing J., Han L., Liu Z., Wang J., Zhang W., Gao A. (2022). Melatonin and probiotics ameliorate nanoplastics-induced hematopoietic injury by modulating the gut microbiota-metabolism. Nano Res..

[10] Zhang Y., Hou B., Liu T., Wu Y., Wang Z. (2023). Probiotics improve polystyrene microplastics-induced male reproductive toxicity in mice by alleviating inflammatory response. Ecotoxicol. Environ. Saf..

[11] Gu S.B., Zhao L.N., Wu Y., Li S.C., Sun J.R., Huang J.F., Li D.D. (2015). Potential probiotic attributes of a new strain of *Bacillus coagulans* CGMCC 9951 isolated from healthy piglet feces. World J. Microbiol. Biotechnol..

[12] Bomko T.V., Nosalskaya T.N., Kabluchko T.V., Lisnyak Y.V., Martynov A.V. (2017). Immunotropic aspect of the *Bacillus coagulans* probiotic action. J. Pharm. Pharmacol..

[13] Lopetuso L.R., Scaldaferri F., Franceschi F., Gasbarrini A. (2016). *Bacillus clausii* and gut homeostasis: State of the art and future perspectives. Expert. Rev. Gastroenterol. Hepatol..

[14] Madempudi R.S., Neelamraju J., Ahire J.J., Gupta S.K., Shukla V.K. (2020). *Bacillus coagulans* Unique IS2 in Constipation: A Double-Blind, Placebo-Controlled Study. Probiotics Antimicrob. Proteins.

[15] Zhai S., Gao Y., Jiang Y., Li Y., Fan Q., Tie S., Wu Y., Gu S. (2024). *Weizmannia coagulans* BC99 affects valeric acid production via regulating gut microbiota to ameliorate inflammation and oxidative stress responses in *Helicobacter pylori* mice. J. Food Sci..

[16] Leung M.C., Williams P.L., Benedetto A., Au C., Helmcke K.J., Aschner M., Meyer J.N. (2008). *Caenorhabditis elegans*: An emerging model in biomedical and environmental toxicology. Toxicol. Sci..

[17] Lenz K.A., Miller T.R., Ma H. (2019). Anabaenopeptins and cyanopeptolins induce systemic toxicity effects in a model organism the nematode *Caenorhabditis elegans*. Chemosphere.

[18] Zhou T., Wu J., Hu X., Cao Z., Yang B., Li Y., Zhao Y., Ding Y., Liu Y., Xu A. (2023). Microplastics released from disposable medical devices and their toxic responses in *Caenorhabditis elegans*. Environ. Res..

[19] Kang Y., Zheng S., Wan T., Wang L., Yang Q., Zhang J. (2023). Nematode as a biomonitoring model for evaluating ecological risks of heavy metals in sediments from an urban river. Ecol. Indic..

[20] Brassea-Pérez E., Hernández-Camacho C.J., Labrada-Martagón V., Vázquez-Medina J.P., Gaxiola-Robles R., Zenteno-Savín T. (2022). Oxidative stress induced by phthalates in mammals: State of the art and potential biomarkers. Environ. Res..

[21] De Marco G., Conti G.O., Giannetto A., Cappello T., Galati M., Iaria C., Pulvirenti E., Capparucci F., Mauceri A., Ferrante M. (2022). Embryotoxicity of polystyrene microplastics in zebrafish *Danio rerio*. Environ. Res..

[22] Waits A., Chen H.C., Kuo P.L., Wang C.W., Huang H.B., Chang W.H., Shih S.F., Huang P.C. (2020). Urinary phthalate metabolites are associated with biomarkers of DNA damage and lipid peroxidation in pregnant women—Tainan Birth Cohort Study (TBCS). Environ. Res..

[23] Zuri G., Karanasiou A., Lacorte S. (2023). Human biomonitoring of microplastics and health implications: A review. Environ. Res..

[24] Hua X., Feng X., Hua Y., Wang D. (2023). Paeoniflorin attenuates polystyrene nanoparticle-induced reduction in reproductive capacity and increase in germline apoptosis through suppressing DNA damage checkpoints in *Caenorhabditis elegans*. Sci. Total Environ..

[25] Gumienny T.L., Lambie E., Hartwieg E., Horvitz H.R., Hengartner M.O. (1999). Genetic control of programmed cell death in the *Caenorhabditis elegans* hermaphrodite germline. Development.

[26] Wang B., Wang Y., Zhang J., Hu C., Jiang J., Li Y., Peng Z. (2023). ROS-induced lipid peroxidation modulates cell death outcome: Mechanisms behind apoptosis, autophagy, and ferroptosis. Arch。 Toxicol..

[27] Brenner S. (1974). The genetics of *Caenorhabditis elegans*. Genetics.

[28] Jiang W., Yan W., Tan Q., Xiao Y., Shi Y., Lei J., Li Z., Hou Y., Liu T., Li Y. (2023). The toxic differentiation of micro- and nanoplastics verified by gene-edited fluorescent *Caenorhabditis elegans*. Sci. Total Environ..

[29] Shang X., Lu J., Feng C., Ying Y., He Y., Fang S., Lin Y., Dahlgren R., Ju J. (2020). Microplastic (1 and 5 μm) exposure disturbs lifespan and intestine function in the nematode *Caenorhabditis elegans*. Sci. Total Environ..

[30] Mueller M.T., Fueser H., Höss S., Traunspurger W. (2020). Species-specific effects of long-term microplastic exposure on the population growth of nematodes, with a focus on microplastic ingestion. Ecol. Indic..

[31] Qu M., Chen H., Lai H., Liu X., Wang D., Zhang X. (2022). Exposure to nanopolystyrene and its 4 chemically modified derivatives at predicted environmental concentrations causes differently regulatory mechanisms in nematode *Caenorhabditis elegans*. Chemosphere.

[32] Schopfer L., Menzel R., Schnepf U., Ruess L., Marhan S., Brümmer F., Kandeler E. (2020). Microplastics effects on reproduction and body length of the soil-dwelling nematode *Caenorhabditis elegans*. Front. Environ. Sci..

[33] Qu M., Qiu Y., Lv R., Yue Y., Liu R., Yang F., Wang D., Li Y. (2019). Exposure to MPA-capped CdTe quantum dots causes reproductive toxicity effects by affecting oogenesis in nematode *Caenorhabditis elegans*. Ecotoxicol. Environ. Saf..

[34] Sun L., Liao K., Wang D. (2020). Comparison of transgenerational reproductive toxicity induced by pristine and amino modified nanoplastics in *Caenorhabditis elegans*. Sci. Total Environ..

[35] Shao Y., Hua X., Li Y., Wang D. (2024). Comparison of reproductive toxicity between pristine and aged polylactic acid microplastics in *Caenorhabditis elegans*. J. Hazard. Mater..

[36] Chen H., Yang Y., Wang C., Hua X., Li H., Xie D., Xiang M., Yu Y. (2022). Reproductive toxicity of uv-photodegraded polystyrene microplastics induced by DNA damage-dependent cell apoptosis in *Caenorhabditis elegans*. Sci. Total Environ..

[37] Corsi A.K., Wightman B., Chalfie M. (2015). A Transparent Window into Biology: A Primer on *Caenorhabditis elegans*. Genetics.

[38] Yu Y., Chen H., Hua X., Dang Y., Han Y., Yu Z., Chen X., Ding P., Li H. (2020). Polystyrene microplastics (PS-MPs) toxicity induced oxidative stress and intestinal injury in nematode *Caenorhabditis elegans*. Sci. Total Environ..

[39] Ferrante M.C., Monnolo A., Del Piano F., Mattace Raso G., Meli R. (2022). The pressing issue of micro- and nanoplastic contamination: Profiling the reproductive alterations mediated by oxidative stress. Antioxidants.

[40] Wang J., Yin J., Peng D., Zhang X., Shi Z., Li W., Shi Y., Sun M., Jiang N., Cheng B. (2025). 4-Nitrophenol at environmentally relevant concentrations mediates reproductive toxicity in *Caenorhabditis elegans* via metabolic disorders-induced estrogen signaling pathway. J. Environ. Sci..

[41] Zhou D., Yang J., Li H., Lu Q., Liu Y.D., Lin K.F. (2016). Ecotoxicological evaluation of low-concentration bisphenol a exposure on the soil nematode *Caenorhabditis elegans* and intrinsic mechanisms of stress response in vivo. Environ. Toxicol Chem..

[42] Kajal S., Thakur S. (2024). Coexistence of microplastics and heavy metals in soil: Occurrence, transport, key interactions and effect on plants. Environ. Res..

[43] Song X., Li C., Qiu Z., Wang C., Zeng Q. (2024). Ecotoxicological effects of polyethylene microplastics and lead (Pb) on the biomass, activity, and community diversity of soil microbes. Environ. Res..

[44] Scuto M., Rampulla F., Reali G.M., Spanò S.M., Trovato Salinaro A., Calabrese V. (2024). Hormetic Nutrition and Redox Regulation in Gut-Brain Axis Disorders. Antioxidants.

[45] Scuto M., Majzúnová M., Torcitto G., Antonuzzo S., Rampulla F., Di Fatta E., Trovato Salinaro A. (2024). Functional Food Nutrients, Redox Resilience Signaling and Neurosteroids for Brain Health. Int. J. Mol. Sci..

[46] Li C., Zhai S., Duan M., Cao L., Zhang J., Wang Y., Wu Y., Gu S. (2024). *Weizmannia coagulans* BC99 Enhances Intestinal Barrier Function by Modulating Butyrate Formation to Alleviate Acute Alcohol Intoxication in Rats. Nutrients.

[47] Gao Y., Li C., Li J., Duan M., Li X., Zhao L., Wu Y., Gu S. (2024). *Weizmannia coagulans* BC99 alleviates hyperuricemia and oxidative stress via DAF-16/SKN-1 activation in *Caenorhabditis elegan*. Front. Microbiol..

[48] Abbas I., Badran G., Verdin A., Ledoux F., Roumie M., Lo Guidice J.M., Courcot D., Garçon G. (2019). In vitro evaluation of organic extractable matter from ambient PM2.5 using human bronchial epithelial BEAS-2B cells: Cytotoxicity, oxidative stress, pro-inflammatory response, genotoxicity, and cell cycle deregulation. Environ. Res..

[49] Kosińska K., Szychowski K.A. (2024). Current state of knowledge of triclosan (TCS)-dependent reactive oxygen species (ROS) production. Environ. Res..

[50] Siddeeg A., AlKehayez N.M., Abu-Hiamed H.A., Al-Sanea E.A., Al-Farga A.M. (2021). Mode of action and determination of antioxidant activity in the dietary sources: An overview. Saudi J. Biol. Sci..

[51] Yang X., Chen J., Liao Z., Xia Z., Huang H., Huang J., Chen L., Fang X., Gao C., Wang J. (2024). *Lactobacillus fermentum* WC2020 increased the longevity of *Caenorhabditis elegans* via JNK-mediated antioxidant pathway. J. Food Sci..

[52] Xie M., Lin L., Xu P., Zhou W., Zhao C., Ding D., Suo A. (2021). Effects of microplastic fibers on *Lates calcarifer* juveniles: Accumulation, oxidative stress, intestine microbiome dysbiosis and histological damage. Ecol. Indic..

[53] Zhang Y., Xie J., Ouyang Y., Li S., Sun Y., Tan W., Ren L., Zhou X. (2024). Adverse outcome pathways of PBDEs inducing male reproductive toxicity. Environ. Res..

[54] Guo X., Xin Q., Wei P., Hua Y., Zhang Y., Su Z., She G., Yuan R. (2024). Antioxidant and anti-aging activities of Longan crude and purified polysaccharide (LP-A) in nematode *Caenorhabditis elegans*. Int. J. Biol. Macromol..

[55] Chen Q., Liu Y., Bi L., Jin L., Peng R. (2024). Understanding the mechanistic roles of microplastics combined with heavy metals in regulating ferroptosis: Adding new paradigms regarding the links with diseases. Environ. Res..

[56] Liu M., Li N., Lu X., Shan S., Gao X., Cao Y., Lu W. (2022). Sweet tea (*Rubus Suavissmus* S. Lee) polysaccharides promote the longevity of *Caenorhabditis elegans* through autophagy-dependent insulin and mitochondrial pathways. Int. J. Biol. Macromol..

[57] Huang J., Liao L., Wang G., Du Z., Wu Z. (2023). Reproductive toxicity of enrofloxacin in *Caenorhabditis elegans* involves oxidative stress-induced cell apoptosis. J. Environ. Sci..

[58] Chen B., Cao X., Lu H., Wen P., Qi X., Chen S., Wu L., Li C., Xu A., Zhao G. (2018). N-(3-oxo-acyl) homoserine lactone induced germ cell apoptosis and suppressed the over-activated RAS/MAPK tumorigenesis via mitochondrial-dependent ROS in *C. elegans*. Apoptosis.

[59] Lettre G., Hengartner M.O. (2006). Developmental apoptosis in *C. elegans*: A complex cednario. Nat. Rev. Mol. Cell Biol..

[60] Yu C., Xu Y., Wei Y., Guo Y., Wang Y., Song P., Yu J. (2024). Gut microbiota and liver metabolomics reveal the potential mechanism of *Lactobacillus rhamnosus* GG modulating the liver toxicity caused by polystyrene microplastics in mice. Environ. Sci. Pollut. Res. Int..

[61] Chen Q., Kong Q., Tian P., He Y., Zhao J., Zhang H., Wang G., Chen W. (2022). Lactic acid bacteria alleviate di-(2-ethylhexyl) phthalate-induced liver and testis toxicity via their bio-binding capacity, antioxidant capacity and regulation of the gut microbiota. Environ. Pollut..

