# Supplementary material for: The Alleviative Effects of Weizmannia coagulans CGMCC 9951 on the Reproductive Toxicity of Caenorhabditis elegans Induced by Polystyrene Microplastics
Source: Microorganisms. 2025 Feb 24;13(3):497. doi: 10.3390/microorganisms13030497 (PMC11944320; doi:10.3390/microorganisms13030497)
Supplement: Supplementary file 1 [file microorganisms-13-00497-s001.zip › microorganisms-3384371-supplementary.pdf]

microorganisms

Table S1. Primer sequences for real-time PCR.

Gene	Forward Primer	Reverse Primer
actin-1	ATGTGTGACGACGAGGTT	GAAGCACTTGCGGTGAAC
hus-1	GCGGCAATCGACGTGTTTAT	CCGGGCAGAACACGTACTAA
clk-2	CACAGTGCCCAACAAAGTCG	TGACATGCTCGCCAGACAAT
cep-1	TACCCGATTCGCAGGACATC	GCATCGGAAATCTTTGGCGT
egl-1	GCCTCAACCTCTTCGGATCT	GCACATTGCTGCTAGCTTGG
ced-3	AGAAGGAGCTTGCTAGAGAGGA	AGTCCTTCGTGTCCCGTAGA
ced-4	TCGAGAGTATCACGTGGATCG	TCTTCGCTTTTTAGCATCAGC
